# Contribution of the EEG in the Diagnostic Workup of Patients with Transient Neurological Deficit and Acute Confusional State at the Emergency Department: The EMINENCE Study

**DOI:** 10.3390/diagnostics15070863

**Published:** 2025-03-28

**Authors:** Maenia Scarpino, Antonello Grippo, Maria Teresa Verna, Francesco Lolli, Benedetta Piccardi, Peiman Nazerian, Patrizia Nencini, Carmela Ielapi, Andrea Nencioni

**Affiliations:** 1 Neurophysiopathology Unit, Careggi University Hospital, 50134 Florence, Italy; scarpinom@aou-careggi.toscana.it (M.S.); ielapic@aou-careggi.toscana.it (C.I.); 2 Emergency Department, Careggi University Hospital, 50134 Florence, Italy; mtverna@gmail.com (M.T.V.); nazerianp@aou-careggi.toscana.it (P.N.); nencionian@aou-careggi.toscana.it (A.N.); 3 Biomedical Science Department, University of Florence, 50121 Florence, Italy; lollif@unifi.it; 4 Stroke Unit, Careggi University Hospital, 50134 Florence, Italy; piccardib@aou-careggi.toscana.it (B.P.); nencinip@aou-careggi.toscana.it (P.N.)

**Keywords:** emergency electroencephalogram, emergency department, transient neurological deficits, acute confusional state diagnostic workup

## Abstract

**Highlights:**

**What are the main findings?**

- An emergent EEG is often required in the differential diagnosis of transient neurological deficit (TND) and acute confusional state (ACS).
- An EEG is useful not only to confirm a diagnostic suspicion but also to help in ruling out initial working diagnoses.

**What is the implication of the main finding?**

- The emergent EEG mainly contributes to a diagnosis when speech disorder, hyposthenia, and ACS are the admission signs/symptoms.
- The emergent EEG shows its greatest usefulness in the final diagnosis of seizures and encephalopathy.

**Abstract:**

**Background/Objectives**: To investigate the usefulness of an emergency electroencephalogram (emEEG) in the differential diagnosis of transient neurological deficits (TND) and acute confusional state (ACS). **Methods**: An analysis was performed on a subset of patients included in EMINENCE, a retrospective study of subjects admitted to the Emergency Department (ED) of our tertiary hospital over a 1-year period. The analysis was limited to patients with neurological symptoms/signs compatible with cerebral hemispheric origin or with an ACS of <24 h duration. We evaluated the usefulness of the emEEG in the diagnostic workup of TND and ACS. **Results**: Speech disorder (75.3%), hyposthenia (68.1%), and ACS (62.9%) were the signs/symptoms with the highest percentage of abnormal emEEGs, especially concerning epileptic discharges. Seizures (85.7%) and encephalopathy (74.3%) were the final diagnoses with the highest percentage of abnormal emEEGs, particularly epileptic discharges and focal slow waves in patients discharged with a diagnosis of seizures, and bilateral slow waves and generalized periodic discharges with triphasic morphology (GPDTM) in patients discharged with a diagnosis of encephalopathy. The presence/absence of epileptic discharges associated with focal slow waves could discriminate between seizures and vascular disease, especially in hyposthenia (100% of seizures when epileptic discharges were present, vs. 50% when absent). Migraine with aura (66%) and an unknown diagnosis (56%) were the final diagnoses with the most normal emEEG. The rapid timing of the emEEG recording compared to the patient’s admission allowed us to perform the test in 29.5% of patients who were still symptomatic, of whom 79% had an abnormal emEEG. **Conclusions**: The emEEG mainly contributed to the diagnosis when speech disorder, hyposthenia, and ACS were the admission signs/symptoms, especially for the final diagnosis of seizures and encephalopathy.

## 1. Introduction

The Emergency Department (ED) is a specific clinical setting where the real challenge is the need to make rapid decisions about the management of patients in order to arrive at the correct diagnosis and, consequently, the correct treatment, often in the absence of the immediate availability of some instrumental tests or of prior specialist consultation.

Transient neurological deficit (TND), a common neurological condition characterized by a focal and transient loss of brain function [1,2], and acute confusional state (ACS), a common clinical neurological/neuropsychiatric disorder with global cognitive impairment and inattention [3,4], usually lead patients to be admitted to the ED and represent two of the most common diagnostic challenges for the attending emergency physician.

The clinical symptom overlap between these different neurological disorders [5,6,7,8,9,10,11,12,13] and their short duration, which often resolves on admission to the ED, with the patient being asymptomatic during the clinical examination, represent the main diagnostic challenges for the emergency physician.

Transient ischemic attack (TIA), a transient episode of neurological dysfunction without acute infarction being visible in diffusion magnetic resonance imaging (MRI) [14], is the neurological disorder that in most cases (80%) underlines a TND [15]. However, focal seizures, migraines with aura [15], or other conditions such as metabolic disorders [16], infections [2], or drug/toxin abuse and psychogenic disorders [17] can be considered as alternative causes of TND.

In addition, both stroke mimics (TNDs of stroke-like presentation, which are not strokes) [2,18,19,20,21,22,23] and stroke chameleons (atypical stroke-like presentations in relation to true strokes) [2,8,12,18,24] need to be considered in patients with TND. In particular, stroke mimics are reported to account for 14.6% of patient admissions to the ED [19].

Finally, ACS is often associated with the same conditions that cause TND, such as non-convulsive status epilepticus (NCSE), vascular disease, sepsis or fever, metabolic or electrolyte disorders, drug/toxin abuse, and psychiatric disorders [13].

According to the literature, targeted investigations such as electrocardiography, laboratory tests, and neuroimaging, especially cerebral magnetic resonance imaging (MRI), are necessary to rule out the various causes of ACS [4,25,26,27]. MRI, which includes diffusion-weighted imaging (DWI), fluid-attenuated inversion recovery imaging (FLAIR), and, in some cases, the gradient-echo sequences associated with cerebral three-dimensional time-of-flight magnetic resonance angiography, is a mainstay of the diagnostic workup for TND of various origins and, for ACS as well, must be considered preferable to brain computed tomography (CT) [2,28,29].

However, in real clinical practice, in a setting such as the ED, especially in a primary-level hospital, but often also in secondary- and tertiary-level hospitals, the attending physicians have to make the correct diagnosis in the absence of the immediate availability of brain MRI. Furthermore, less than half (39%) of patients with TIA, the most common cause of TND and one of the main causes of ACS, show detectable lesions with DWI [30,31].

These limitations lead to the widespread use of alternative diagnostic tools in clinical practice, such as the electroencephalogram (EEG), a non-invasive and inexpensive instrumental test that could be performed at the patient’s bedside, but this is in the absence of universally accepted guidelines [32,33] and in the absence of strong evidence in the literature on the contribution made by an emergent EEG (emEEG) in the differential diagnosis of TND or ACS.

The diagnostic power of the EEG in the differential diagnosis of TND has been analyzed in only one previous study [2], which reported no difference in EEG patterns between TIA, seizure, stroke mimic, stroke chameleon, and other diagnoses, while Prud’hon et al. [13] analyzed the role of EEG in the diagnostic workup of patients with ACS, reporting that this neurophysiological test was a key investigation tool in the management strategy of ACS in 11% of patients admitted to the ED.

However, both of these previous studies had some limitations, mainly related to the small number of patients analyzed and the timing of EEG recording, with median delays of 1.6 and 1.5 days after symptom onset, respectively.

In the current study, we report the emEEG patterns of a large sample of consecutive patients admitted to the ED of a tertiary care hospital for TND or ACS, with the emEEGs performed early after symptom onset.

The main aim of our study was to observe the frequency and characteristics of emEEG abnormalities in patients with different neurologic admission symptoms and with different final diagnoses of TND and ACS, and to observe the contribution of emEEGs to the differential diagnosis of TND/ACS.

## 2. Materials and Methods

### 2.1. The EMINENCE Study

The present investigation is based on the analysis of a subset of patients from the monocentric retrospective EMINENCE (electroencephalograms in Emergency Departments) study [34] performed at the Careggi University Hospital, Florence, Italy.

The methodology of EMINENCE has been described in detail elsewhere [34]. Briefly, we retrospectively evaluated all patients older than 18 years, admitted to the ED, who underwent an emEEG between 1 January 2023 and 31 December 2023.

The main objective of the EMINENCE study was to determine the reason why ED physicians ordered an emEEG and its benefits, defined as changes in the clinical diagnosis, the diagnostic–therapeutic management of patients, and their subsequent discharge or hospitalization.

Clinical data were obtained from medical records. Both demographic and clinical variables were included in the analysis. Discharge or the hospitalization of patients was also reported. Finally, we recorded EEGs performed both during and outside normal hospital hours. We included requests for a second EEG only if they were still requested by the ED.

In the present study, we only included in our analysis those patients with neurological symptoms or signs compatible with a cerebral hemispheric origin (speech disorders, motor, sensory, or visual symptoms) or with an ACS, all with a duration of <24 h [2,13]. We kept those patients who had any of these previous symptoms associated with the presence of headaches as a separate group. We reported the time of symptom onset compared to the emEEG recording and its duration. Finally, we reported whether the symptoms were still present during the EEG recording.

### 2.2. EmEEG Recording and Classification

EmEEGs were available at our institution during normal hospital hours (8:00 to 19:00 from Monday to Friday and 8:00 to 14:00 on Saturday). EEGs were also available by telephone 24 h a day outside normal hospital hours for emergencies related to the detection of SE or NCSE.

Standard 30-min EEG recordings were obtained using a digital machine and a prewired EEG head-cap with 19 electrodes (Fp1-Fp2-F7-F8-F3-F4-C3-C4-T3-T4-P3-P4-T5-T6-O1-O2-Fz-Cz-Pz), positioned according to the 10–20 international standard system. The recordings were made at a sampling rate of 128 Hz. During the review, digital filters (low-pass filter = 30 to 70 Hz; time constant = 0.1 or 0.3 s; notch filter = 50 Hz) and sensitivity gain (2 to 10 µV/mm, with a standard gain of 7 µV/mm) were adjusted according to interpretation needs [35,36,37].

EEG recordings were usually performed in the absence of drug sedation. EEG recordings were classified according to the American Clinical Neurophysiology Society (ACNS) EEG terminology [38] by expert neurologists/neurophysiologists as soon as possible after the attending physician’s request, depending on the rest of the daily work activity, but usually giving priority to requests from the ED. The EEG descriptors considered were continuity, voltage, the organization of an anterior-posterior gradient of background activity, the presence of reactivity and spontaneous variability of background activity, frequency, symmetry, the presence of epileptic discharges, slow waves, periodic patterns, patterns with triphasic morphology, and the presence of detectable transient sleep patterns in the EEG. Finally, the presence of patterns indicative of seizures or NCSE was also reported. For further details of EEG descriptors, see Hirsch et al. [38].

### 2.3. Final TND/ACS Classification

Final TND and ACS classification between “ischemic” [2,14,39], “focal seizure” [40,41], and “migraine with aura” [42] was established according to the classical criteria [1,2,13]. Final TND and ACS etiologies were defined as “encephalopathies” when metabolic or electrolyte disorders, fever or septic state, and the introduction or withdrawal of drugs or substances were involved [2,13]. We used the term “other” when TND and ACS could not be classified in the previous categories but had a defined aetiology, such as minor head trauma, focal brain lesion, and psychiatric disorder [6,13,43,44,45,46]. Finally, we defined TND/ACS with an undetermined aetiology or a non-neurological cause as “unknown” [13].

### 2.4. Decision-Making Criteria

The final diagnosis was made at the patient’s discharge from the ED on the basis of clinical characteristics and instrumental data by emergency physicians, assisted by neurologists with expertise in neurovascular disorders, epileptic disorders, and migraine. Patients’ symptoms were classified according to the most likely diagnosis, based on the clinician’s expertise, and because other diagnoses were excluded.

### 2.5. Outcome Assessment

Correlation analysis was performed between the presence of abnormal emEEG patterns and clinical and demographic data. In addition, emEEG patterns were analyzed and compared between the final groups on discharge from the ED. Finally, the usefulness of the emEEG was assessed on the basis of the subsequent clinical management of the patient, including the contribution of the emEEG to the final diagnosis.

### 2.6. Statistical Analysis

Our results were reported as numbers and percentages for categorical variables and as medians (interquartile range: 25th percentile to 75% percentile) for quantitative variables. We used descriptive statistics to define our population and compared data between the groups using chi-squared tests. To avoid overestimating associations, we used Cramer V tests for categorical correlations. For Cramer V results, values between 0.30 and 0.49 were considered a good association, while values above 0.50 were considered a strong association. The statistical software used was Jamovi (version 2.6.2), two-tailed tests were used, and *p*-values of <0.05 were considered significant. The Bonferroni method was used to correct multiple analyses.

### 2.7. Standard Protocol Approvals, Registrations, and Patient Consent

The study complied with ethical principles and good clinical practice guidelines. Due to logistical constraints, informed consent is not possible for all patients. A retrospective analysis of the database in which the patients were enrolled was approved by the local ethics committee (Ethics Committee Area Vasta Centro 27241, positive opinions of the Ethics Committee number 27241_oss).

## 3. Results

In the present secondary analysis of data from the EMINENCE monocentric retrospective study, we included 603 emEEGs performed on 579 patients out of a total of 1018 patients recruited in the first analysis between 1 January 2023 and 31 December 2023. During their stay in the ED, 24 patients underwent a second EEG. Most emEEGs (*n* = 600; 99.5%) were performed during working hours.

The median age of the cohort was 73 years (IQR 24). Two hundred and ninety patients (49.2%) were female.

In total, 124 subjects (21.0%) had a history of epilepsy, 47 had unknown aetiology, and 77 had structural aetiology. One hundred and six subjects (18.3%) were taking anti-seizure medication (ASM).

Three hundred and ninety-two patients (67.7%) had no previous brain parenchymal damage. Of the remaining 187 patients, 69 (36.8%) had undergone a previous neurosurgical intervention. The most common causes of brain damage were multi-infarct encephalopathy (135 patients; 23.3%), previous ischemic stroke (46 patients; 7.9%), brain tumor (24 patients; 4.1%), hemorrhagic stroke (10 patients; 1.8%), and previous traumatic brain injury (7 patients; 1.2%).

In total, 119 patients (20.5%) were admitted to the ED with metabolic or electrolyte disorders, 141 patients (24.3%) were found to have fever or sepsis, and 13 patients (2.2%) were admitted after drug abuse.

Brain CT scans were performed in 558 patients (96.4%) and showed acute pathology in 90 patients (15.2%), in whom cerebral ischemia was the most common form of brain damage (14 patients; 2.4%).

Twenty-four patients (3.8%) underwent a lumbar puncture, of whom two received results suggestive of central nervous system (CNS) infection.

The demographic characteristics of the patients are shown in Table 1.

In total, 208 patients (35.9%) were admitted to the ED for ACS, of whom 247 (42.6%) had a speech disorder, 69 (11.9%) had motor symptoms, 24 (4.1%) had sensory symptoms, 26 patients (4.5%) had headaches associated with one of these other neurological symptoms, and 5 patients (0.8%) were admitted with visual symptoms.

The median duration of symptoms was 60 min (IQR 345). Two hundred and three patients (35.1%) still had the neurological symptoms that led to their admission to the ED at the time of their Emergency Department visit.

The median delay from symptom onset to EEG recording was 350 min (5–6 h) (IQR 675 min).

In total, 171 patients (29.5%) had neurological symptoms that led to their admission to the ED when the emEEG was performed, and 135 of them (79%) had an abnormal neurophysiological test. Of these, 24 patients (17.7%) underwent a second EEG during their stay in the ED. More specifically, 1 patient showed NCSE, not detected in the first recording, 1 patient showed generalized periodic discharges with triphasic morphology (GPDTM), in 9 patients, an improvement in the emEEG was detected, and in the last 13 patients, the emEEG was unchanged. The remaining 408 patients (70.4%) showed the remission of neurological signs and symptoms when the emEEG was performed, with 252 subjects (61.7%) providing an abnormal neurophysiological test.

The demographic characteristics of the patients included previous neurosurgical intervention (*n* = 61 of 69 patients, 88.4%), sepsis (*n* = 58 of 66 patients, 87.0%), metabolic disorder (*n* = 33 of 39 patients, 84. 6%), previous brain parenchymal damage without previous neurosurgery (*n* = 152 of 187 patients, 81.2%), electrolyte disturbance (*n* = 64 of 80 patients, 80.0%), and fever (*n* = 52 of 65 patients, 80.0%), all of which were the most common predisposing factors for an abnormal EEG.

According to the patients’ signs and symptoms on admission, we found an abnormal EEG in 186/247 patients with speech disorder (75.3%), in 47/69 patients with hyposthenia (68.1%), in 131/208 patients with ACS (62.9%), in 14/26 patients with headache (53.8%), in 2/5 patients with visual symptoms (40%), and in 8/24 patients with hypoesthesia (33.3%). The distribution of abnormal emEEGs according to symptom presentation tended to be significant (Cramer’s V = 0.156).

Table 2 shows a comprehensive list of emEEG characteristics and abnormalities, shown according to neurological signs and symptoms on admission.

**Figure 1:**
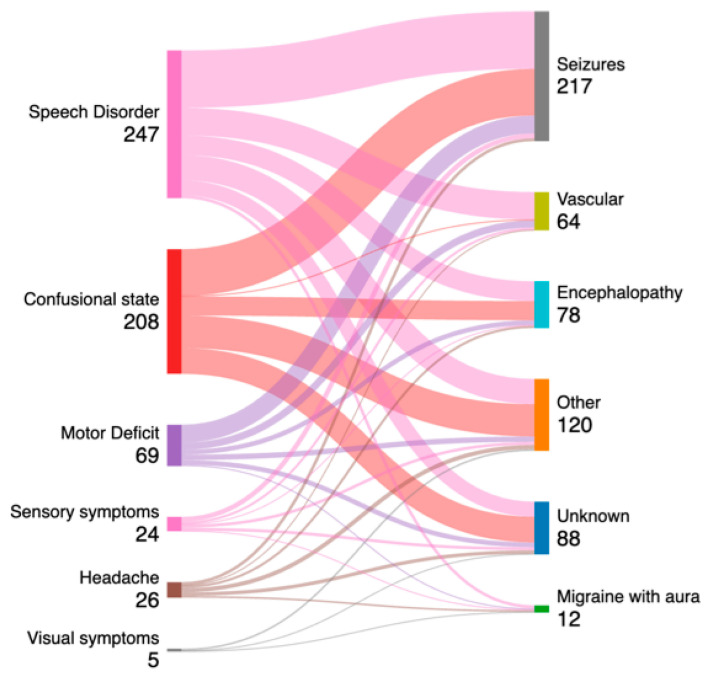
Sankey diagram showing the relationship between the discharge diagnosis groups and the neurological signs and symptoms on admission to the ED recorded in patients who underwent emEEGs.

An alluvial plot showing the discharge diagnosis, compared with the neurological signs and symptoms on admission to the ED, in patients who underwent emEEGs is shown in Figure 1.

According to the alluvial plot, 217 patients were discharged with a seizure, along with 64 with a vascular disorder, of whom 47 subjects were discharged with a TIA, 14 with an atypical stroke [2], and 3 with a rapidly regressive ischemic attack (RRIA) when an acute infarct was detected on MRI diffusion sequences [2]. In total, 78 patients had a final diagnosis of encephalopathy, of which cases 43 were due to sepsis, 14 to metabolic disturbance, 7 to electrolyte disturbance, 12 to substance abuse, and 2 to non-specific fever. One hundred and twenty patients were discharged in the “other” group, which included minor head trauma, focal brain lesions, and psychiatric disorders as the causes of the neurological symptomatology that brought the patients to the ED. In addition, 12 patients were discharged with migraine with aura and the remaining 88 subjects were discharged as part of the “unknown” group if the TND/ACS had no defined aetiology or non-neurological cause.

According to the different final diagnoses, we found abnormal EEGs in 186/217 patients with seizures (85.7%), in 58/78 patients with encephalopathy (74.3%), in 39/64 patients with vascular disease (60.9%), in 62/120 patients with other diagnoses (52.1%), in 38/88 patients with unknown diagnoses (43.1%), and in 4/12 patients with migraine with aura (33.3%). The distribution of abnormal emEEGs according to final diagnoses was significant (Cramer’s V = 0.26).

Migraine with aura (8/12 patients; 66%) and unknown diagnoses (50/88 patients; 56%) were the final diagnoses with the most normal emEEGs.

Table 3 shows a comprehensive list of emEEG characteristics and abnormalities, shown according to the final diagnosis of the patient.

Table 4 shows the number of each abnormal emEEG feature according to the different final diagnoses of the most common neurological signs or symptoms on admission.

The emEEG influenced the initial diagnostic suspicion in 452 (78.1%) patients, confirming the initial diagnosis in 93 patients (16.1%) and ruling it out in 319 (55.1%), in association with anamnestic, clinical, and other instrumental data (usually, a brain CT scan).

In total, 185 patients (32%) underwent subsequent hospitalization, 3 (0.5%) refused hospitalization, and the remaining 391 (67.5%) were discharged home from the ED. In addition, 191 patients (33%) presented an EEG within normal limits, and 157 of them (82.1%) were discharged home within 24 h of admission to the ED.

## 4. Discussion

According to our results, speech disorder (75.3%), hyposthenia (68.1%), and ACS (62.9%) were the neurological signs/symptoms recorded on admission to the ED that showed the highest percentage of abnormal emEEG readings, especially when epileptic discharges were taken into account. Seizures (85.7%) and encephalopathy (74.3%) were the final diagnoses with the highest percentage of abnormal emEEGs, particularly epileptic discharges and focal slow waves in patients discharged with a diagnosis of seizures and bilateral slow waves and GPDTM in patients discharged with a diagnosis of encephalopathy, regardless of the admission symptoms. The presence/absence of epileptic discharges associated with focal slow waves discriminated between seizures and vascular aetiology, especially for the admission symptom of hyposthenia (100% seizures when epileptic discharges were present vs. 50% when focal slow waves alone were present). Instead, migraine with aura (66%) and unknown diagnosis (56%) were the final diagnoses with the most normal emEEGs.

The rapid timing of the emEEG recording compared to the patient’s admission to the ED (median delay 5–6 h) allowed us to perform neurophysiological tests in 29.5% of patients who were still symptomatic, of whom 79% had an abnormal emEEG.

Transient neurological deficits and ACS are usually diagnostic challenges, especially for ED physicians who must make rapid decisions about patient management.

A brain MRI is the instrumental test that can best identify part of the differential diagnosis of ACS and especially of TND and must be considered preferable to a brain CT scan [28].

However, in real clinical practice, especially in a setting such as the ED, clinicians must make the correct diagnosis without the immediate availability of this instrumental test, also bearing in mind, however, that only less than half (39%) of patients with TIA, this being the most common cause of TND and one of the main causes of ACS, have detectable lesions on DWI [30,31]. These limitations have led to the widespread use of alternative instrumental tests, such as the emEEG, a non-invasive and inexpensive diagnostic tool that can be performed at the patient’s bedside.

The EEG, which allows functional exploration of the brain, is considered complementary to neuroimaging in the detection of different neurological conditions such as NCSE or encephalopathy of different aetiology, and its usually rapid availability, even in an emergency context, has led to the wide use of this diagnostic tool in the ED, but this is used in the absence of universally accepted guidelines [32,33] and in the absence of strong evidence in the literature on the contribution of emEEGs to the differential diagnosis of TND and ACS.

In fact, only a few previous studies [2,13,17,47,48,49] have analyzed the diagnostic power of emEEGs in patients with TND or ACS.

More specifically, only Prud’hon et al. [13] analyzed patients with ACS and reported that the emEEG was a key examination stage in the management strategy of ACS in 11% of patients admitted to the ED, with limitations regarding the small sample size and the long delay between symptom onset and neurophysiological test recording (mean 1.5 days).

Regarding the studies on TND, three of them [47,48,49] considered a single aetiology of TND. The study by Seo-Young Lee et al. [47] analyzed post-traumatic TND, and Ji Hoon Phi et al. [48] reported data on post-operative TND, while Madkour et al. [49] analyzed patients with TIAs.

In another study [17], Vellieux et al. analyzed the role of spectral EEG analysis in the differential diagnosis of TND and reported a discriminative EEG power in migraine with aura compared to TND of other origins. However, in an emergency setting such as the ED, where clinicians need information quickly to make prompt decisions about patient management, spectral analysis could not be used as an emergency tool since it requires more time to perform compared to a normal interpretation of emEEG results according to ACNS EEG terminology [38]. In addition, this study had other limitations related to the small sample being analyzed and the timing of the emEEG recording, including patients with TND symptom onset within the previous seven days.

At the same time, Madkour et al. [49] also reported the discriminative power of EEG spectral analysis compared to a conventional EEG in a small sample of patients with TIA.

To date, the only previous study analyzing the diagnostic power of conventional emEEGs in the differential diagnosis of TND reported no difference in EEG patterns between TNDs of different origins [2]. The authors reported that the EEG was abnormal in 42% of cases and that focal slow waves were the most common finding. However, this EEG pattern was found in all diagnostic groups; therefore, it did not allow an etiological orientation. However, this previous study also had limitations related to the small number of patients analyzed and the timing of the EEG recording, with a median delay of 1.6 days after symptom onset, suggesting that a delayed EEG may be less informative than an emEEG performed in the first few hours. A study by Lorenzon et al. [2] showed the same limitations as the paper by Vellieux et al. in 2021 [17], as both studies were performed on the same sample of patients.

In the current study, we recruited all consecutive patients admitted to the ED of our hospital who fulfilled the inclusion criteria and who underwent emEEG, including both neurophysiological tests recorded during daily clinical practice and those tests performed outside hospital hours.

The power of the present study was mainly represented by the large sample analyzed and the rapid timing of the EEG execution compared to the symptom onset, with a median recording time of 350 min (between 5 and 6 h), which allowed us to perform the neurophysiological test when the patient was still symptomatic in 29.5% of subjects, a result very different from previous studies [2,13], and which allowed us to obtain a very high percentage of abnormal EEGs: 79% were from patients who were still symptomatic, and 61.7% were from patients with resolution of the neurological symptoms on admission.

Another strength of our study was the inclusion of patients with both TND and ACS in the analysis. Indeed, the frequent overlapping of clinical symptoms between these different neurological disorders [5,6,7,8,9,10,11,12,13] and the assumption that the underlying etiologies are often the same can hinder the correct diagnostic workup, especially in the ED setting, where attending physicians usually have to make rapid decisions about the diagnostic–therapeutic management of patients, often in the absence of the immediate availability of some instrumental tests, such as brain MRI, or prior neurological or neurosurgical consultation.

Speech disorder, ACS, and hyposthenia were the presenting neurological signs/symptoms with both a high emEEG demand and a high percentage of abnormal emEEGs, especially when epileptic discharges were taken into account. More specifically, seizures, periodic discharges, or epileptic discharges were never reported in patients with visual symptoms and were reported in only one patient for both sensory disturbances and a headache associated with speech disorder. This result is important because an emEEG is usually requested to confirm an epileptic suspicion as the cause of symptoms upon access to the ED and, according to our data, hyposthenia, speech disorder, and ACS were the presenting neurological signs/symptoms for which emEEG was most useful when a seizure was considered in the differential diagnosis.

At the same time, seizure was the most commonly reported final diagnosis for all these three most frequent admission symptoms, followed by vascular disease for speech disorder and hyposthenia.

Although encephalopathy was not one of the most frequent final diagnoses in patients with these three preceding neurological signs/symptoms, this was the discharge diagnosis most associated with an abnormal emEEG after seizure, especially for ACS and speech disorder. In particular, encephalopathy was the only final diagnosis, other than seizures, in which the emEEG could show a specific abnormal pattern, the GPDTM, which allowed clinicians not only to exclude an epileptic origin of TND/ACS but also to suggest an alternative aetiology.

According to our results, 37.4% of all patients were discharged with seizures, 13% with encephalopathy, and 11% with vascular disease. These last two percentages were very similar, despite the fact that while encephalopathy is a diagnosis that can be detected by emEEG, this neurophysiological test should not be the gold standard for detecting vascular disease. The main explanation for this similar percentage of discharge aetiology could be the atypical presentation of some vascular diseases, as observed in our results (21.8%), characterized by the march of symptoms, multiple stereotyped episodes, isolated speech disorder, tingling sensations, or rigid postures. This percentage may seem high compared to the diagnosis of TIA (70%), but it must be borne in mind that the present sample of patients is limited to those who underwent an emEEG and in whom an epileptic aetiology was, therefore, suspected.

Seizures were the final diagnosis in which epileptic discharges were most frequently reported, with a percentage of 37.7%, a result in line with the previous literature [50,51,52], and their presence contributed significantly to the final diagnosis of seizures, regardless of the admission symptom (hyposthenia, speech disorder, or ACS). According to our results, the focal slow-wave EEG pattern was also significantly associated with a final diagnosis of seizures when speech disorder and ACS were the admission symptoms. In these cases, we believe that a second EEG may almost be necessary to consider these EEG changes as an expression of a post-critical state, rather than their being related to another aetiology such as vascular disease.

With regard to vascular disease, we also reported that it was the second most common final diagnosis after a seizure when hyposthenia and speech disorder were the presenting symptoms. In these cases, the presence/absence of epileptic discharges associated with focal slow waves allowed us to discriminate between the two aetiologies of seizures and vascular disease, especially for hyposthenia (100% of seizures when epileptic discharges were present, vs. 50% when focal slow waves alone were present).

Overall, the analysis of our results showed that emEEGs influenced the initial diagnostic suspicion in 452 (78.1%) patients, confirming the initial diagnosis in 93/579 (16.1%) and ruling it out in 319/579 (55.1%), in association with anamnestic, clinical, and other instrumental data (usually, a brain CT scan). This is a result of great clinical importance because, in a specific clinical setting such as that of the ED, where the real challenge is the need to make rapid decisions about the management of patients, often in the absence of the immediate availability of some instrumental tests or of prior specialist consultation, not only the confirmation but also the ruling out of the initial diagnosis could be of great importance in achieving the correct diagnosis and, consequently, the correct treatment.

Finally, migraine with aura was the final diagnosis with the most normal emEEG (66%), followed by “unknown origin” (56%), characterized by a non-neurological aetiology causing TND/ACS or by an undefined aetiology. Regarding the admission signs/symptoms, although we are aware of the preliminary nature of our results, given the very small sample of patients with visual disturbance, we reported that none of them had epileptic discharges and none of them was discharged with a diagnosis of seizure. With regard to sensory disturbance and the group of patients who also had headaches, only one patient had an epileptic discharge diagnosis for both symptoms, compared with eight and five patients discharged with seizure diagnoses, respectively. These results suggested that the usefulness of the emEEG could be limited when these three previous admission signs/symptoms were present or when migraine with aura and “unknown origin” as suspected diagnoses were concerned. This is an important point, particularly for primary and secondary hospitals, where the availability of emEEG is limited and resources are scarce, along with the need for highly qualified staff.

Our study has some limitations. The main limitation of our study is the classification of the patients. An emEEG was often requested to confirm or exclude epileptic seizures as the cause of the neurological symptoms presenting at the ED, or if the patients who had an emEEG were those in whom a diagnosis of TND or confusional state was uncertain. In addition, although experts in vascular disorders, epilepsy, and migraine participated in the classification, multiple aetiologies were possible and some patients may have been misclassified, especially because our evaluation is limited to discharge from the ED and we have no subsequent outpatient neurological follow-up. In particular, 88 patients remained without a diagnosis at the end of their medical care. However, we did not find a better classification method than the combination of clinical, biochemical, and instrumental data, and this represents the daily challenge of emergency physicians. Another limitation concerns the ability of a routine 30-min emEEG (such as that used in our clinical practice in patients admitted to the ED) to record interictal epileptic discharges or even non-convulsive seizures [52]. According to the literature, a 30-min emEEG does indeed fail to record approximately 47–50% of epileptic discharges [50,51,52].

In addition, given the retrospective nature of the research, clinical and instrumental information was collected from medical records and may have been incomplete.

Although our sample of patients was significantly larger than those in previous studies, we were able to include a limited number of subjects with certain presentation signs/symptoms (sensory and visual disturbances or symptoms associated with headache), making our results preliminary, at least for this category of patients.

Furthermore, this study was conducted in a tertiary hospital where at least two or three neurophysiological technicians and one EEG expert neurologist/neurophysiologist per day were exclusively dedicated to recording and interpreting emEEGs during their working hours. We are aware that, in clinical practice, this daily work organization has made it possible both to reduce the time taken to perform emEEGs after patient admission and to cope with the large number of requests for emEEGs, many of which came from the ED department, and, thus, to collect the current, large sample of patients. We also recognize that this daily work organization could not be extended to primary and secondary hospitals, where the availability of emEEGs is limited. In addition, our sample of patients, who were recruited in a tertiary teaching hospital, included mainly complex and severe cases that may not fully represent the wider spectrum of cases in smaller hospitals, limiting the generalizability of our work.

In conclusion, this study, which included a large sample of subjects with a rapid timing of EEG performance compared to symptom onset and which was limited to emEEGs performed in the ED, was mainly helpful in establishing the final diagnosis of seizures and encephalopathy, especially when speech disorder, hyposthenia, and ACS were the presenting neurological signs/symptoms. In addition, the presence/absence of epileptic discharges associated with focal slow waves allowed differentiation between seizures and vascular etiologies, especially regarding the admission symptom of hyposthenia. Migraine with aura and “unknown diagnosis” were instead the final diagnoses with the most normal emEEGs. Further multicenter prospective studies with large samples, especially for some of the admission signs/symptoms such as sensory and visual disturbances and headache, and studies restricting the evaluation of emEEGs performed in the ED setting are needed to increase the robustness and generalizability of our findings. However, these preliminary results should encourage clinicians to request an emEEG as soon as possible, mainly after the onset of speech disturbance, hyposthenia, and ACS, especially if this is of short duration.

## Figures and Tables

**Table 1:** Patient demographic characteristics.

Patients, *n*	579
Age year, median (IQR)	73 (IQR 24)
Female gender, *n* (%)	290 (49.2%)
Previous epileptic seizures	124 (21.0%)
Unknown aetiology	47 (8.1%)
Structural aetiology	77 (13.3%)
Antiseizure medication	106 (18.3%)
Under-dosed antiseizure medication	22 (3.8%)
Fever	65 (11.2%)
Sepsis	76 (12.9%)
Metabolic disturbance	39 (6.7%)
Electrolyte disturbance	80 (13.8%)
Drug abuse	13 (2.2%)
Previous neurological history	388 (67.0%)
Stroke	54 (9.1%)
Neurosurgery	69 (13.9%)
Cardiac disorders	114 (19.7%)
Diabetes	105 (18.1%)
Dyslipidemia	154 (26.6%)
Thyroid disease	69 (11.9%)
Brain computer tomography	558 (96.4%)
Recent brain computed tomography lesions	90 (15.2%)
Lumbar puncture	24 (3.8%)
Positive	2 (8.3%)
Home discharge	391 (67.5%)
Hospitalization	185 (32.0%)
Hospitalization refused	3 (0.5%)

**Table 2:** EmEEG characteristics and abnormalities, shown according to the most frequent neurologic signs/symptoms on admission.

	Epileptic Discharges/ Seizures	Major Focal Waves	Major Bilateral Waves	Generalized Periodic Discharges with Triphasic Morphology (GPDTM)
Cramer’s V	0.12	0.14	0.13	0.07
**Initial Symptoms on Admission**				
Speech Disorder (*n* = 247)	42 (17.0%)	117 (47.3%)	117 (47.3%)	3 (1.2%)
Acute Confusional State (*n* = 208)	31 (14.9%)	57 (27.4%)	68 (32.6%)	5 (2.4%)
Motor deficit (*n* = 69)	16 (23.1%)	30 (43.4%)	30 (43.4%)	1 (1.4%)
Headache (*n* = 26)	1 (3.8%)	8 (30.7%)	3 (11.5%)	1 (3.8%)
Sensory deficit (*n* = 24)	1 (4.1%)	6 (25.0%)	3 (11.5%)	0 (0.0%)
Visual disorder (*n* = 5)	0 (0.0%)	0 (0%)	1 (20%)	0 (0.0%)

**Table 3:** Comprehensive list of emEEG characteristics and abnormalities, shown according to the final diagnosis of the patient.

	Epileptic Discharges/ Seizures	Major Focal Waves	Major Bilateral Waves	Generalized Periodic Discharges with Triphasic Morphology (GPDTM)
Cramer’s V	0.46	0.25	0.15	0.14
**Final Diagnosis**				
Seizures (*n* = 217)	82 (37.7%)	129 (59.4%)	115 (52.9%)	4 (1.8%)
Vascular Disease (*n* = 64)	1 (1.5%)	24 (37.5%)	20 (31.2%)	0 (0%)
Migraine with aura (*n* = 12)	0 (0%)	3 (25.0%)	1 (8.3%)	0 (0%)
Encephalopathies (*n* = 78)	5 (6.4%)	15 (19.2%)	49 (62.8%)	6 (7.6%)
Other (*n* = 120)	1 (0.8%)	31 (25.8%)	37 (30.8%)	0 (0.0%)
Unknown (*n* = 88)	3 (3.4%)	17 (19.3%)	27 (30.6%)	0 (0.0%)

**Table 4:** The number of each abnormal emEEG feature (bold type), shown according to the different final diagnoses of the most common neurological signs or symptoms on admission.

Final Diagnosis	Seizures	Encephalopathies	Vascular Disease	Migraine with Aura	Other	Unknown
**Initial Symptoms on Admission**						
**Epileptic discharges/ Seizures**						
Speech Disorder (*n* = 247)	38/96	2/33	1/46	0/5	1/42	0/25
Acute Confusional State (*n* = 208)	26/78	3/32	0/1	0/0	0/54	2/43
Motor deficit (*n* = 69)	15/30	0/8	0/12	0/2	0/9	1/8
**Major Focal Waves**						
Speech Disorder (*n* = 247)	71/96	9/33	18/46	1/5	14/42	4/25
Acute Confusional State (*n* = 208)	35/78	4/32	0/1	0/0	8/55	10/43
Motor Deficit (*n* = 69)	17/30	2/8	4/12	1/2	3/9	3/8
**Major Bilateral Waves**						
Speech Disorder (*n* = 247)	55/96	24/33	17/46	1/5	23/42	4/25
Acute Confusional State (*n* = 208)	40/78	22/32	2/2	0/0	10/55	19/43
Motor deficit (*n* = 69)	2/30	5/8	3/12	0/2	3/9	1/8
**Generalized Periodic Discharges ** **with Triphasic Morphology** **(GPDTM)**						
Speech Disorder (*n* = 247)	1/96	2/33	0/46	0/5	0/42	0/25
Acute Confusional State (*n* = 208)	3/78	2/32	0/1	0/0	0/54	4/43
Motor Deficit (*n* = 69)	0/30	1/8	0/12	0/2	0/9	0/8

## Data Availability

The data presented in this study are available on request from the corresponding author.
